# Network meta-analysis of the efficacy of physical exercise interventions on vision health in children and adolescents

**DOI:** 10.3389/fpubh.2024.1393909

**Published:** 2024-08-29

**Authors:** Jianming Liu, Wencen Lan, Danxuan Zhang

**Affiliations:** ^1^The School of Physical Education and Sports, Central China Normal University, Wuhan, China; ^2^The School of Physical Education, Dali University, Yunnan, China

**Keywords:** children, adolescents, vision health, myopia, physical activity, network meta-analysis

## Abstract

**Objective:**

This study systematically evaluates the impact of different physical exercise modalities on vision health interventions for Chinese children and adolescents.

**Methods:**

A comprehensive search was conducted in databases, including Web of Science, PubMed, EBSCO, MEDLINE, Embase, and CNKI. The focus was on randomized controlled trial (RCT) studies related to physical activity interventions for vision health in this demographic. The search covered literature from the inception of each database until May 1, 2023. Two researchers independently conducted literature screening, data extraction, and risk of bias assessment, adhering to pre-established inclusion and exclusion criteria. A network meta-analysis was performed using the “Network” package in Stata 14.2.

**Results:**

The analysis encompassed 17 studies with 1,840 participants aged 7 to 18 years. Findings from the network meta-analysis revealed that badminton [MD = 0.23 (0.12, 0.33), *p* < 0.001] and table tennis [MD = 0.16 (0.09, 0.22), *p* < 0.001] exercises, along with health education [MD = 0.13 (0.03, 0.23), *p* = 0.013], were statistically significant in enhancing vision health compared to no intervention. According to the Surface Under the Cumulative Ranking (SUCRA) probability ranking, badminton (SUCRA = 96.7) and table tennis (SUCRA = 84.1) emerged as the most effective modalities for myopia intervention in children and adolescents, with health education (SUCRA = 73.2) following closely.

**Conclusion:**

Physical exercise significantly contributes to the healthy development of vision in children and adolescents. Among various activities, badminton and table tennis are the most effective in improving visual health, highlighting the need for augmented promotion of visual health education. However, the quantity and quality of the included studies necessitate further high-quality intervention research to confirm these findings.

## 1 Introduction

In recent years, the global incidence of myopia has been steadily rising, with a notable surge among children and adolescents (1, 2). Projections indicate that by 2050, nearly half of the world’s population may be affected by varying degrees of myopia, with the prevalence among Chinese youth potentially exceeding 80% (3–6). The COVID-19 pandemic has further exacerbated this trend, as children and adolescents increasingly rely on electronic devices for extended periods for online learning (7, 8). The impairment of vision and the discomfort and pain caused by the damage to visual functions can lead to various psychological issues, including anxiety, worry, depression, and melancholy (9–11). Individuals with visual dysfunction often experience anxiety and tension in their daily lives due to the difficulty in perceiving their surroundings. Myopia patients may feel frustrated because they cannot see things clearly, leading to negative emotional experiences. The accumulation of long-term psychological stress may lead to the onset of depression, causing individuals to fall into states of emotional lowness and loss of interest. This emphasizes the critical impact of vision health on the physical development of China’s youth, necessitating urgent attention to finding effective strategies to mitigate myopia in this demographic.

Physical activity is known to play a crucial role in bolstering the physical health of young people and mitigating the spread of myopia (12–14). Research indicates that higher levels of physical activity correlate with a reduced risk of myopia development and a slower progression of the condition (15), Additionally, children with myopia tend to engage less in high-intensity physical activities (16). In essence, physical activity serves as a significant deterrent against myopia (17). The study (18) reviews the relationship between physical activity in children and adolescents and myopia and points out, there is no correlation between the duration of physical activity in children and adolescents with myopia and the progression of myopia, and it suggests that high-quality intervention studies are needed to verify its conclusions. Based on this, some independent studies have indicated that engaging in outdoor physical activities can reduce the incidence of myopia among children and adolescents (19). At the same time, there are also studies suggesting that yoga exercises can effectively prevent myopia in adolescents and improve symptoms of poor vision (20, 21). With the continuous increase in the number of intervention studies, research on the impact of physical activity on the progression of myopia in children and adolescents has entered the stage of systematic review. The study (22) conducted a meta-analysis of eight independent studies on the impact of table tennis on visual health and concluded that the physical exercise program of table tennis has a good effect on improving vision. Another study (23) pointed out through a meta-analysis of 30 independent studies that net sports can play a protective and preventive role in the visual health of children and adolescents against myopia. The sports interventions included in the study are table tennis, badminton, gymnastics, soccer, basketball, middle and long-distance running, and sports games, among others. Another meta-analysis (24) concluded through subgroup analysis of 11 studies that outdoor activities, table tennis, and badminton have significant results in improving the vision of primary and secondary school students. It can be seen that the impact of physical exercise on visual health is unquestionable, but the specific methods of physical exercise intervention have not been clarified. The comparative impact of various exercise forms and identifying the most beneficial type of physical exercise for myopia in children and adolescents remains unclear (1). Therefore, exploring more scientific and efficient physical activity methods to improve visual health in this demographic is a vital and ongoing area of research.

In this study, we employed different physical activity modalities as interventions for myopia in children and adolescents, conducting a comprehensive meta-analysis. Our goal was to assess and rank the effectiveness of these various physical activity modalities in managing myopia. This analysis aims to identify the optimal physical activity modality, providing a reference for devising precise exercise programs tailored to prevent myopia in young individuals and to enhance their overall physical development.

## 2 Methodology of the study

### 2.1 Inclusion and exclusion criteria

For this study, we adhered to the PRISMA (Preferred Reporting Items for Systematic Reviews and Meta-Analyses) guidelines to systematically evaluate the impact of physical activity on visual health (25, 26). Our inclusion and exclusion criteria were established based on the “PICOS” framework detailed in these guidelines, covering P (study population), I (intervention), C (comparator), O (outcome indicator), and S (study design).

#### 2.1.1 Study population (P)

This included children and adolescents aged 7 to18 years, enrolled in primary and secondary schools across China (27).

#### 2.1.2 Interventions (I)

We considered various physical activity modalities, ranging from individual sports to comprehensive physical activity programs.

#### 2.1.3 Comparator (C)

Comparisons were made either with no intervention or with controlled studies involving non-physical activity interventions, such as health education programs.

#### 2.1.4 Outcome indicator (O)

The primary outcome was naked eye vision (static vision) assessed using the Standard Logarithmic Visual Acuity Scale (SLVAS). We included both bilateral naked eye vision and separate assessments of left and right-eye vision.

#### 2.1.5 Study type (S)

Eligible studies included randomized controlled trials (RCTs) and non-randomized controlled studies focusing on physical activity interventions for visual health in children and adolescents.

#### 2.1.6 Exclusion criteria

Studies were excluded if: ① the full text was not accessible; ② the study population did not consist of the specified age group (7 to 18 years) or included individuals with pathological eye diseases; ③ the literature was in languages other than Chinese or English.

### 2.2 Literature search strategy

To ensure a comprehensive literature review, three researchers independently conducted searches across multiple databases, including Web of Science, PubMed, EBSCO, Embase, MEDLINE, and CNKI. The search spanned from the inception of each database to May 1, 2023, providing a thorough exploration of existing literature.

**Figure 1:**
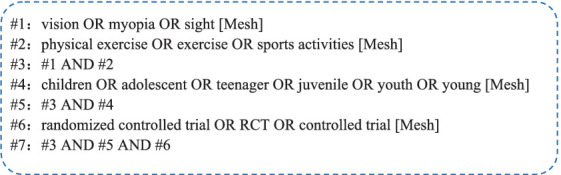
Search strings used to search the PubMed database.

To enhance the study’s reliability and validity of our study, we implemented a meticulous primary literature search. Our search terms were extensive and included, encompassing both Chinese and English keywords. In Chinese, terms such as physical exercise, physical activities, sports, children, adolescents, vision, vision health and myopia were utilized. In English corresponding terms such as physical exercise, exercise, sports activities, children, adolescent, juvenile, vision, myopia, sight, youth, young, teenager were included. Compound search terms, as illustrated in Figure 1, were also employed.

Furthermore, we included references from the selected studies and relevant literature recommended by the databases, ensuring a comprehensive and scientifically rigorous search approach.

### 2.3 Literature screening and data extraction

Literature screening and data extraction were meticulously conducted by two researchers, strictly adhering to the predetermined inclusion and exclusion criteria. In cases of disagreement or inconsistency in the extracted data, discussions were facilitated with a third researcher to achieve a consensus. The extracted information included: ① Authors’ names, year of publication, and region.② Characteristics of the subject population, including age and sample size. ③ Details of interventions, such as the period, duration, and frequency; ④ A seven-point risk of bias evaluation. ⑤ Mean values, standard deviations, and standard errors of the outcome indicators for both the intervention and control groups in each study.

### 2.4 Literature quality assessment of included studies

The methodological quality of the included studies was appraised using the Physiotherapy Evidence Database (PEDro) scale, consisting 11 items. Items 2 to 11 being scored out of a possible 10 points. Studies scoring ≥6 were categorized as high quality, those with scores between 4 and 5 as moderate quality, and scores <4 as low quality. The quality assessment was independently conducted by two researchers, with any scoring discrepancies resolved through discussion with a third researcher.

### 2.5 Statistical analysis

We conducted a network meta-analysis using the “Network” package in Stata 14.2 software (28, 29), employing a frequentist approach. Visual acuity assessments, measured using a lightbox-type “E” shaped vision chart, were considered as continuous variables in the included studies. The mean difference (MD) served as the effect size, with a 95% confidence interval (CI) used for effect size evaluation. A MD’s 95% CI not including 0 was indicative of a statistically significant difference between interventions (equivalent to *p* < 0.05).

For inconsistency testing, nodal analysis was employed, where a *p*-value <0.05 suggested inconsistency between direct and indirect comparisons (30). Conversely, a *p*-value >0.05 indicated good consistency. Additionally, we utilized the node-splitting method for local inconsistency testing.

The study results were summarized using the Surface Under the Cumulative Ranking (SUCRA) indicator. A larger area under the curve denoted more effective interventions. SUCRA values, presented as percentages ranging from 0 to 100%, reflected the efficacy of a particular intervention, with higher percentages indicating greater efficacy (31).

## 3 Results

### 3.1 Literature screening process and results

**Figure 2:**
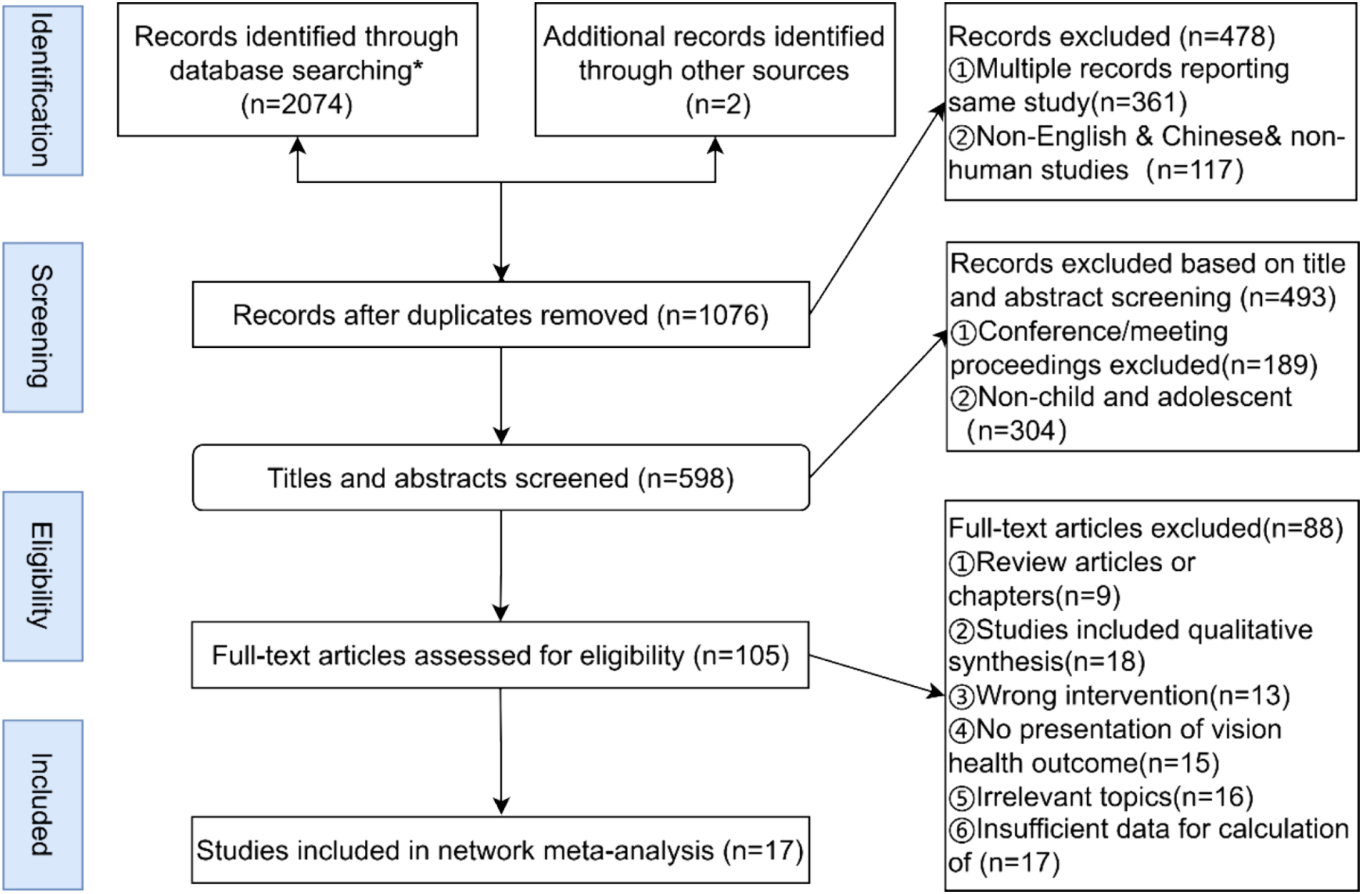
Literature screening process. *Results of specific searches in each database PubMed (*n* = 381), EBSCO (*n* = 327), web of science (*n* = 327), CNKI (*n* = 351), MEDLINE (*n* = 387), Embase (*n* = 301).

We implemented our developed search strategy across six major international and domestic databases, resulting in the identification 2074 literature pieces relevant to our study’s theme. Additionally, two more pertinent articles were uncovered through alternative means. To streamline this extensive dataset, we utilized EndNote X9 software to remove duplicates and initiated an initial screening. Subsequently, we meticulously reviewed titles and abstracts to further assess relevance. A thorough examination of the full texts followed, allowing us to exclude any literature that did not meet our predefined inclusion and exclusion criteria. This rigorous process ultimately led to the selection of seventeen studies for our network meta-analysis. A detailed illustration of the search process and the results of the literature selection can be found in Figure 2.

### 3.2 Basic characteristics of included studies

In this study, we selected seventeen studies for the network meta-analysis, involving a total of 1,840 participants aged between 7 to 18 years. Each study had an intervention period of at least 8 weeks. The primary outcome measure across all studies was naked eye vision (static vision). The fundamental characteristics of these studies are detailed in Table 1. Notably, three studies employed a three-arm experimental design, while the remaining utilized a two-arm approach.

**Table 1:** Basic characteristics of included studies.

Studies	Samples (T/C)	Age (Mean ± SD)	Intervention (T/C)	Period (w)	Frequency	Min/Time	Outcome measurement
Guo et al. (32)	Pupil (30/30)	12.9 ± 0.9	Table tennis/No treatment	104	3	120	Static visual acuity
You (33)	Pupil (26/26)	No mention (4, 5 grade)	Table tennis/Gym class	16	2	60	Static visual acuity
Wang and Liu (34)	Pupil (60/60)	11 ~ 18	Table tennis/No treatment	24	3	60	Static visual acuity
Zhang and Li (35)	Pupil *(30/30)	No mention	Table tennis/No treatment	10	4	60	Static visual acuity
Zhang (36)	Pupil *(30/30)	7 ~ 8	Table tennis/No treatment	12	3	60	Static visual acuity
Song et al. (37)	Pupil (96/96)	9 ~ 18	Table tennis/Gym class	Long>8	4	90	Static visual acuity
Nie (38)	Pupil (93/92)	11 ~ 14	Sports game/ Gym class	16	3	20–40	Static visual acuity
Wang et al. (19)	Pupil (89/88)	9.19 ± 1.37	Sports activity/Gym class	36	4–6	60	Static visual acuity
Xiao (39)	Pupil *(30/30)	7 ~ 8	Table tennis/Middle-distance race	16	3	60	Static visual acuity
Chen et al. (40)	Pupil (30/30)	No mention (4grade)	Table tennis/Gym class	16	3	40	Static visual acuity
Hong (41)	Pupil (25/25)	No mention	Table tennis/No treatment	80	3	120	Static visual acuity
Hu (42)	Pupil (40/40/40)	No mention	Table tennis/Middle-distance race/Basketball#	80	4	90+	Static visual acuity
Cai et al. (43)	Pupil (82/76)	7.25 ± 0.44–10.24 ± 0.43	Dynamic vision/No treatment	16	3	40	Static visual acuity
Zhou (44)	Pupil (30/30)	T:8.03 ± 0.67\u00B0C:8.03 ± 1.42	Sports game/Gym class	16	4	40	Static visual acuity
Dai and Ye (21)	Pupil *(35/35/35)	8 ~ 9	Badminton/Education/No treatment #	40	3–5	90	Static visual acuity
Zhang (45)	Pupil *(20/20/20)	13 ~ 15	Badminton/Education/No treatment #	8	3	60	Static visual acuity
Zhang (46)	Pupil (43/36)	7 ~ 8	Dynamic vision/Gym class	60	4	20–30	Static visual acuity

T: represents the intervention group in the studies. C: denotes the control group. Min: minutes, used in the context of intervention duration. *: Indicates studies where the intervention group included participants with pseudomyopia. Mean ± SD: Stands for mean ± standard deviation, a statistical measure used to convey the average result and variability of the data. #: Symbolizes studies that employed a three-arm experimental design, offering a broader comparative analysis.

The selected studies featured a diverse range of intervention modalities, including ball sports like table tennis, badminton, and basketball, track and field activities with a focus on long-distance running, and various related physical activities. These interventions encompassed physical games, sports activities, physical education classes (Gym class), dynamic visual physical activities, as well as health education and non-intervention controls.

Specifically, ‘sports games’ referred to activities that stimulate physical engagement, such as the game “tearing name tags.” ‘Gym class’, a mandatory course in China’s compulsory education system, aims to impart basic physical education and health care knowledge, develop students’ skills and athletic abilities, and contribute to their ideological and moral education. ‘Sports activities’ included various formats, either organized or informal, intended to provide relaxation and enjoyment for students. ‘Dynamic Visual Physical Activity’ involved integrating visual cognitive tasks into physical exercises, requiring participants to identify and report visual markers (such as pictures or letters) while engaged in sports like soccer, basketball, or track and field.

### 3.3 Literature quality assessment

The quality of the 17 studies included in our network meta-analysis underwent rigorously assessment. The average quality score for these papers was 6.12, indicating an overall high standard of literature quality. For detailed quality assessment results for each study, please refer to Table 2.

**Table 2:** Assessment of quality of literature.

Studies	1	2	3	4	5	6	7	8	9	10	11	PEDro sum score
Guo et al. (32)	1	1	1	1	1	0	0	1	1	0	1	7
You (33)	1	1	0	1	0	0	0	1	1	1	0	5
Wang and Liu (34)	1	1	1	0	0	1	0	1	1	1	1	7
Zhang and Li (35)	1	1	1	1	1	0	0	0	1	0	1	6
Zhang (36)	1	1	0	1	1	0	0	1	1	1	1	7
Song et al. (37)	1	1	1	0	0	0	0	1	1	1	1	6
Nie (38)	1	1	1	1	1	0	0	1	1	0	1	7
Wang et al. (19)	1	1	0	1	1	1	0	1	1	1	0	7
Xiao (39)	1	1	1	1	0	0	0	1	1	1	1	7
Chen et al. (40)	1	0	0	1	1	0	0	1	1	1	1	7
Hong (41)	1	1	0	0	0	0	0	1	1	1	1	5
Hu (42)	1	1	0	1	0	0	0	1	1	1	0	5
Cai et al. (43)	1	1	0	1	1	0	0	1	1	0	1	6
Zhou (44)	1	1	1	1	0	0	0	1	1	1	1	7
Dai and Ye (21)	1	1	0	1	0	0	0	1	1	1	0	5
Zhang (45)	1	0	0	1	0	0	0	1	1	1	1	5
Zhang (46)	1	0	0	1	0	0	0	1	1	1	1	5

Criteria: 1: eligibility criteria specified; 2: subjects randomly allocated to groups; 3: concealed allocation; 4: groups were similar at baseline; 5: blinding of all subjects; 6: blinding of al therapists; 7: blinding of all assessors; 8: measures obtained from more than 85% of subjects allocated to groups; 9: subjects received treatment or control condition as allocated, or intention-to-treat analysis; 10: between-group statistical comparisons reported for at least one outcome; 11: both point measures and measures of variability were reported. 0: high risk of bias; 1: low risk of bias, sum score is sum of “1” count per reference.

### 3.4 Network of included studies

**Figure 3:**
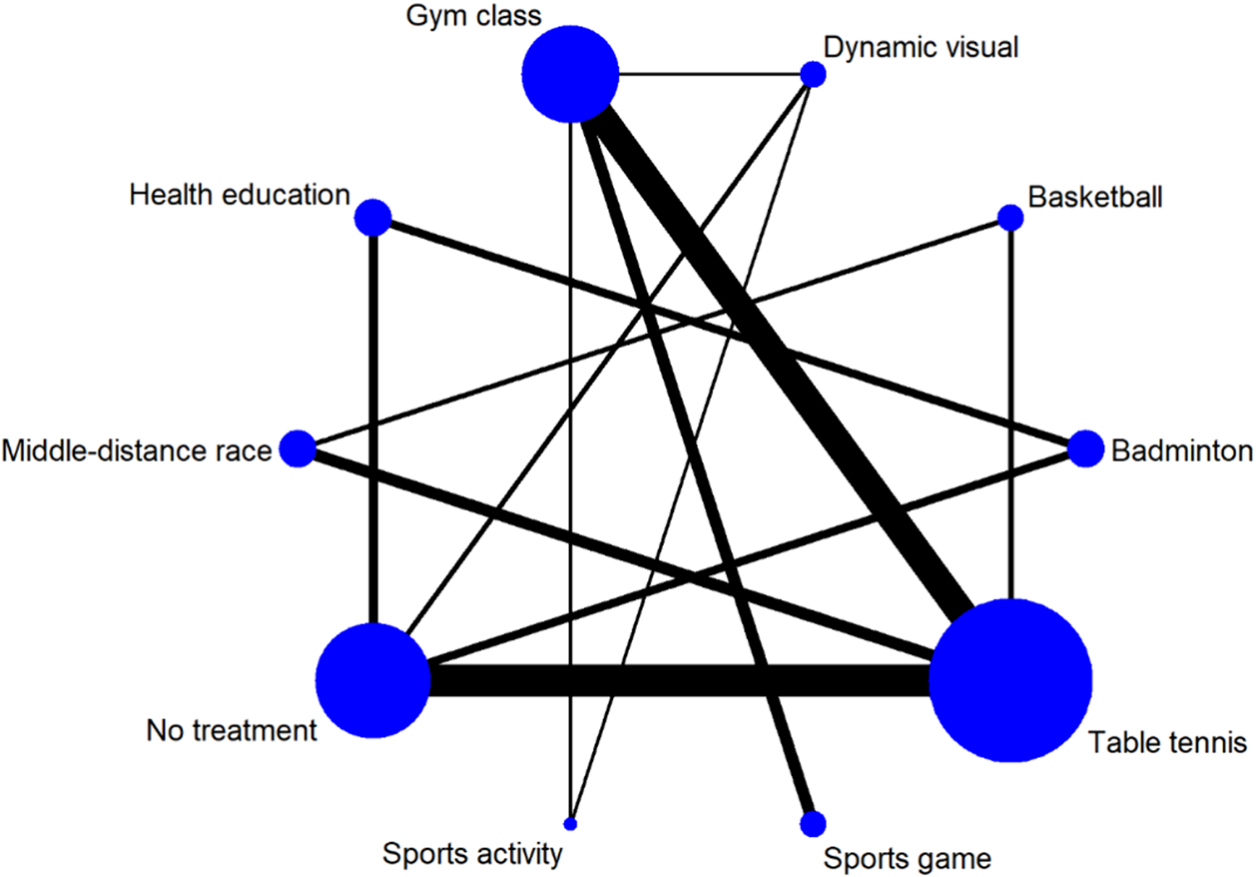
Meshwork of different physical activity modalities for myopia intervention in children and adolescents. Dynamic vision: dynamic vision sports activity.

Figure 3 illustrates the network of various physical activity modalities employed in myopia interventions for children and adolescents. In this graphical representation, the size of each node correlates with the sample size of each intervention type, where larger nodes indicate larger sample sizes. Furthermore, the thickness of the lines connecting the nodes signifies the number of studies that have compared the respective interventions. Consequently, thicker lines denote a greater number of comparative studies between two specific interventions.

### 3.5 Results of network meta-analysis

#### 3.5.1 Inconsistency test

The nodal analysis model yielded a *p*-value of 0.83 (>0.05), indicating no significant inconsistency. This suggests that the results of both direct and indirect comparisons of the effectiveness of various interventions for myopia in children and adolescents are consistent, allowing for analysis using the consistency model.

#### 3.5.2 Results of consistency analysis

The consistency analysis revealed that interventions such as table tennis [MD = 0.16, 95% CI (0.09, 0.22), *p* = 0.000], badminton [MD = 0.23, 95% CI (0.12, 0.33), *p* = 0.000], and health education [MD = 0.13, 95% CI (0.03, 0.23), *p* = 0.013] significantly outperformed no intervention, demonstrating notable effectiveness in vision health interventions for children and adolescents. Conversely, interventions like middle and long-distance running [MD = 0.06, 95% CI (−0.05, 0.18), *p* = 0.301], sports games [MD = 0.03, 95% CI (−0.10, 0.16), *p* = 0.681], basketball [MD = 0.07, 95% CI (−0.22, 0.07), *p* = 0.317], general physical activity [MD = 0.17, 95% CI (−0.14, 0.17), *p* = 0.832], and kinesthetic physical activity [MD = 0.10, 95% CI (−0.14, 0.21), *p* = 0.085], as well as regular physical education classes [MD = 0.001, 95% CI (−0.09, 0.09), *p* = 0.990], did not demonstrate significant effectiveness for vision health in children and adolescents.

#### 3.5.3 Local inconsistency

The local inconsistency test, utilizing the node-splitting method, showed that all interventions had *p*-values greater than 0.05, signifying no local inconsistency. This confirms that the direct and indirect comparison results of each intervention are in alignment.

### 3.6 Ranking of network meta-analysis

The ranked results of the network meta-analysis interventions are displayed in Table 3. A higher SUCRA (Surface Under the Cumulative Ranking) percentage indicates greater intervention effectiveness. The rankings for different physical activities on myopia interventions in children and adolescents were as follows: badminton (SUCRA = 96.7) > table tennis (SUCRA = 84.1) > health education (SUCRA = 73.2) > kinesthetic physical activity (SUCRA = 65.2) > middle and long-distance running (SUCRA = 51.1) > sports games (SUCRA = 37.6) > general physical activities (SUCRA = 33.7) > regular physical education classes (SUCRA = 25.6) > basketball (SUCRA = 25.3) > no intervention (SUCRA = 7.5).

**Table 3:** SUCRA values for effectiveness of each intervention.

Intervention	SUCRA (%)	PrBest	MeanRank	Ranking
Badminton	95.3	75.2	1.4	1
Table tennis	81.4	10.8	2.7	2
Health education	74.8	5.5	3.3	3
Dynamic visual	64.1	4.7	4.2	4
Middle-distance race	48.5	0.9	5.6	6
Sports game	34.7	0.4	6.9	7
Gym class	22.6	0	8.0	8
Sports activity	50.4	2.5	5.5	5
Basketball	20.1	0	8.2	9
No treatment	8.2	0	9.3	10

Comparative results for each intervention, based on SUCRA rankings, along with effect sizes and 95% confidence intervals (MD + 95% CI) for all comparisons, are presented in Table 4. A 95% CI not including 0 indicates significant comparisons.

**Table 4:** League table reporting the comparative effects for all interventions for the vision health network (MD + 95%CI).

Badminton	Table tennis	Health education	Dynamic visual	Middle-distance race	Sports game	Sports activity	Gym class	Basketball	No treatment
0.07 (−0.06,0.20)									
0.10 (−0.01,0.20)	0.03 (−0.10,0.15)								
0.13 (−0.03,0.28)	0.06 (−0.06,0.17)	0.03 (−0.12,0.18)							
**0.16 (0.01,0.32)**	**0.09 (0.00,0.19)**	0.07 (−0.08,0.22)	0.04 (−0.11,0.19)						
**0.20 (0.03,0.37)**	**0.13 (0.01,0.24)**	0.10 (−0.06,0.27)	0.07 (−0.08,0.23)	0.03 (−0.12,0.18)					
**0.21 (0.02,0.40)**	0.14 (−0.01,0.29)	0.11 (−0.07,0.3-)	0.08 (−0.06,0.23)	0.04 (−0.13,0.22)	0.01 (−0.17,0.19)				
**0.23 (0.09,0.36)**	**0.15 (0.10,0.21)**	0.13 (−0.00,0.26)	0.10 (−0.02,0.22)	0.06 (−0.05,0.17)	0.03 (−0.07,0.13)	0.02 (−0.13,0.16)			
**0.23 (0.12,0.33)**	**0.16 (0.09,0.22)**	**0.13 (0.03,0.23)**	0.10 (−0.01,0.21)	0.06 (−0.05,0.18)	0.03 (−0.10,0.16)	0.02 (−0.14,0.17)	0.00 (−0.09,0.09)		
**0.30 (0.12,0.48)**	**0.23 (0.10,0.35)**	**0.20 (0.20,0.38)**	**0.17 (0.00,0.34)**	**0.13 (0.01,0.26)**	0.10 (−0.07,0.27)	0.09 (−0.11,0.29)	0.07 (−0.06,0.21)	0.07 (−0.07,0.22)	

The bold part is significant for pairwise comparison.

### 3.7 Publication bias test

**Figure 4:**
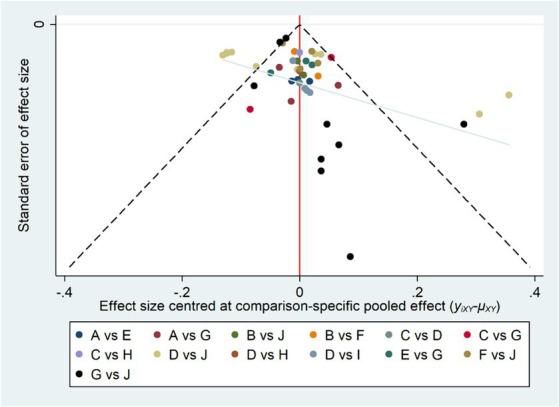
Comparison-adjusted funnel plot for myopia intervention effects.

To assess the presence of publication bias in the studies included in our analysis, we employed comparison-adjusted funnel plots (47). These plots utilized the effect of myopia intervention as the key effect indicator. As depicted in Figure 4, each point on the plot represents an individual study. The observed symmetry in the funnel plots suggests a lower likelihood of publication bias and small-sample effects among the included literature. This symmetry indicates a balanced and unbiased representation of study results, underscoring the reliability of our meta-analytical findings (see Figure 4).

## 4 Discussion

The primary aim of this study was to assess the impact of physical exercise on myopia intervention in children and adolescents through using a systematic review and network meta-analysis approach. The objective was to identify the most effective physical exercises for managing myopia in this demographic. The findings underscore the significant role of physical exercise in myopia intervention for children and adolescents, aligning with related studies (18). The most beneficial forms of physical activity identified for myopia intervention were badminton (95.3%) and table tennis (81.4%), with health education (74.8%) also deemed critical.

### 4.1 Effectiveness of physical exercise in vision health interventions

Myopia occurs when the axial length of the eye surpasses the focal length of the cornea and lens, leading to refractive error (48). Prolonged near vision and sustained focus are primary contributors to vision impairment (49). Continuous near vision induces strain on the ciliary muscles, potentially leading to spasms and pseudomyopia. If left unaddressed, this can accelerate the onset of myopia (49). Extended periods of near vision in children and adolescents can cause sustained contraction and eyestrain, hindering the ability of ciliary muscles to relax (50). Maintaining normal ciliary muscle function and alleviating eyestrain are effective strategies for preventing and controlling myopia.

Physical exercise serves as an active rest mechanism after prolonged near vision activities, alleviating eyestrain, relaxing the ciliary muscles, preventing pseudomyopia, and improving blood circulation around the eyes (51). Various physical exercise modalities, such as basketball (42), middle-distance running (39), and yoga (20), have proven effective in intervening in myopia development in children and adolescents.

The “light-dopamine” hypothesis supports the effectiveness of outdoor physical activities in mitigating myopia. The intensity of light significantly impacts visual acuity. Animal studies show that that visual deprivation leads to reduced retinal dopamine content (52–54). Bright light stimulates retinal dopamine synthesis and release, inhibiting eye axis growth (55, 56), thereby contributing to myopia prevention. This finding supports studies highlighting the effectiveness of outdoor physical exercises, such as comprehensive outdoor activities (19) and sports games (20), in preventing myopia in children and adolescents.

### 4.2 Effectiveness of badminton and table tennis exercises in vision health interventions for children and adolescents

The network meta-analysis, comparing nine different interventions directly and indirectly, revealed that badminton (95.3%) and table tennis (81.4%) exercises possess a significant advantage in myopia intervention for children and adolescents. Myopia development is intricately linked to ciliary muscle function, and dysregulation of these muscles can cause blurred retinal imaging, subsequently leading to myopia (57). Badminton and table tennis exercises not only mitigate eye fatigue and prevent the onset of pseudomyopia but also enhance ciliary muscle regulation more effectively than other sports exercises. These activities require rapid shifts between far and near vision, training the ciliary muscles to contract and relax swiftly, thereby enhancing their sensitivity and precision.

Existing research on improving myopia through ball sports is also mainly focused on small ball games such as table tennis and badminton, and there are relevant explanations in the mechanism research of improving visual health. In these types of sports, the ball moves quickly and changes direction frequently, requiring participants to closely watch the trajectory of the ball to make correct judgments. This provides excellent exercise for the adjustment of the eye’s refractive power and the contraction of the ciliary muscles. Compared to large ball sports, badminton and table tennis have relatively smaller ranges and amplitudes of physical activity, but the frequency of stimulation the eyes receive per unit of time is relatively higher. Large ball sports involve a larger range of physical activity, and large balls (such as basketballs and soccer balls) take relatively longer to roll with each movement, resulting in a relatively lower frequency of stimulation to the eyes per unit of time. This is also why relevant studies have shown that large ball sports (such as basketball, etc.) (42) have a certain effect on improving myopia, but compared to small ball sports, the effect is not significant. During badminton and table tennis play, the varying speeds and directions of the ball necessitate vigilant tracking and accurate judgment by the eyes. This dynamic environment fosters the training of alternating distant and near vision. When following the fast-moving ball, the ciliary muscles contract, relaxing the lens suspensory ligament, allowing the lens to increase its curvature and refractive index due to its elasticity. Conversely, when returning the ball, the process reverses. The long-range shots also aid in relaxing the ciliary muscle, alleviating any spasms. Thus, these sports exercises not only bolster blood circulation and metabolism in the eyes, alleviating fatigue, but also provide comprehensive training of the ciliary muscle, enhancing its regulatory ability.

Furthermore, badminton and table tennis exercises are advantageous over other sports due to their minimal requirements for venues, personnel, and technical skill levels. They enable children and adolescents to engage more readily and deeply in the exercise experience, thereby effectively improving ciliary muscle regulation.

### 4.3 Research limitations and prospects

The review found that there are some shortcomings in the existing studies on the intervention of physical exercise in the visual health of children and adolescents: (1) Most research consists of cohort and cross-sectional studies, with fewer dedicated intervention studies, leading to varied findings. Future research should address potential differences in confounding variables for a more accurate exercise prescription. (2) Studies have often conflated the effects of “physical exercise” with “outdoor activities.” Future studies should distinguish the specific influences of “outdoor factors” and “physical activity” in myopia management. Additionally, most research have focused on naked-eye visual acuity as the primary outcome measure, there is a paucity of research on dynamic visual acuity and related physiological indexes is needed. (3) The quality of independent intervention studies needs enhancement. Applying “blinding” techniques, implementing high-quality intervention studies, and controlling extraneous variables are crucial for scientifically robust evidence.

This review also has some limitations and deficiencies: (1) This study primarily focuses on the effects of physical exercise on visual health within the intervention period, but in reality, long-term follow-up data on participants would provide more compelling evidence for the sustained impact of physical activity on the visual health development of children and adolescents. (2) The included physical exercises encompass specific sports, outdoor activities, and physical training, and different types of physical exercises as well as the intensity of physical activity may all lead to content bias. (3) The number of studies on sports interventions for the progression of myopia in children and adolescents is relatively small, and there is an imbalance in the number of studies on different sports activities, which means that the conclusions drawn from the network meta-analysis require further validation by more high-quality research. (4) The data obtained all come from published literature, and some studies lack extractable data, which limits the number of studies included in the network meta-analysis. These deficiencies all need to be further explored and addressed in future research.

From the study results, it’s evident that, alongside physical activity, health education is pivotal intervention for the visual health of children and adolescents. Combining health education with physical activity interventions holds promise for future research in myopia interventions. The presence of publication bias and small sample effects may influence outcomes, requiring further high-quality intervention studies for validation.

## 5 Conclusion

The study’s findings highlight the effectiveness of physical activity as an intervention for enhancing the visual health of children and adolescents. Among various physical activities, badminton and table tennis stand out as the most beneficial modalities for achieving this purpose. Additionally, the study emphasizes the importance of enhancing education and awareness regarding visual health. Such efforts are crucial for raising awareness among children and adolescents about the importance of safeguarding their visual well-being.

However, the quality of the literature reviewed in this research underscores the need for additional high-quality intervention studies to validate and strengthen these conclusions. Future research endeavors should aim to provide more substantial and reliable evidence, further confirming the efficacy of physical activities in addressing visual health issues among children and adolescents.

## Data availability statement

The original contributions presented in the study are included in the article/supplementary material, further inquiries can be directed to the corresponding author.

## Author contributions

WL: Investigation, Conceptualization, Data curation, Methodology, Software, Writing – original draft. JL: Investigation, Writing – original draft, Writing – review & editing. DZ: Conceptualization, Investigation, Data curation, Methodology, Writing – review & editing.
